# Vasopressin and cardiovascular autonomic adjustment in chronic hypertensive pregnancy

**DOI:** 10.1038/s41440-024-01769-6

**Published:** 2024-07-22

**Authors:** Mirjana Jovanović, Bojana Stevanović, Vladislav Pajović, Tatjana Tasić, Maja Lozić, Ljiljana Đukić, Marija Kosić, David Murphy, Nina Japundžić-Žigon

**Affiliations:** 1 Department of Pathophysiology, University of Belgrade Faculty of Medicine, Belgrade, RS Serbia; 2 University of Belgrade Faculty of Dentistry, Belgrade, RS Serbia; 3 Department of Pharmacology, University of Belgrade Faculty of Medicine, Belgrade, RS Serbia; 4 Bristol Medical School: Translational Health Sciences, University of Bristol, Bristol, UK

**Keywords:** Autonomic cardiovascular control, Chronic hypertensive pregnancy, Vasopressin, Paraventricular nucleus, Supraoptic nucleus

## Abstract

Chronic hypertensive pregnancy (CHP) is a growing health issue with unknown etiology. Vasopressin (VP), a nonapeptide synthesized in paraventricular (PVN) and supraoptic nucleus (SON), is a well-known neuroendocrine and autonomic modulator of the cardiovascular system, related to hypertension development. We quantified gene expression of VP and its receptors, V1aR and V1bR, within the PVN and SON in CHP and normal pregnancy, and assessed levels of secreted plasma VP. Also, we evaluated autonomic cardiovascular adaptations to CHP using spectral indices of blood pressure (BPV) and heart rate (HRV) short-term variability, and spontaneous baroreflex sensitivity (BRS). Experiments were performed in female spontaneously hypertensive rats (SHRs) and in normotensive Wistar rats (WRs). Animals were equipped with a radiotelemetry probe for continuous hemodynamic recordings before and during pregnancy. BPV, HRV and BRS were assessed using spectral analysis and the sequence method, respectively. Plasma VP was determined by ELISA whilst VP, V1aR, and V1bR gene expression was analyzed by real-time-quantitative PCR (RT-qPCR). The results show that non-pregnant SHRs exhibit greater VP, V1aR, and V1bR gene expression in both PVN and SON respectively, compared to Wistar dams. Pregnancy decreased VP gene expression in the SON of SHRs but increased it in the PVN and SON of WRs. Pregnant SHRs exhibited a marked drop in plasma VP concentration associated with BP normalization. This triggered marked tachycardia, heart rate variability increase, and BRS increase in pregnant SHRs. It follows that regardless of BP normalization in late pregnancy, SHRs exhibit cardiovascular vulnerability and compensate by recruiting vagal mechanisms.

**Figure:**
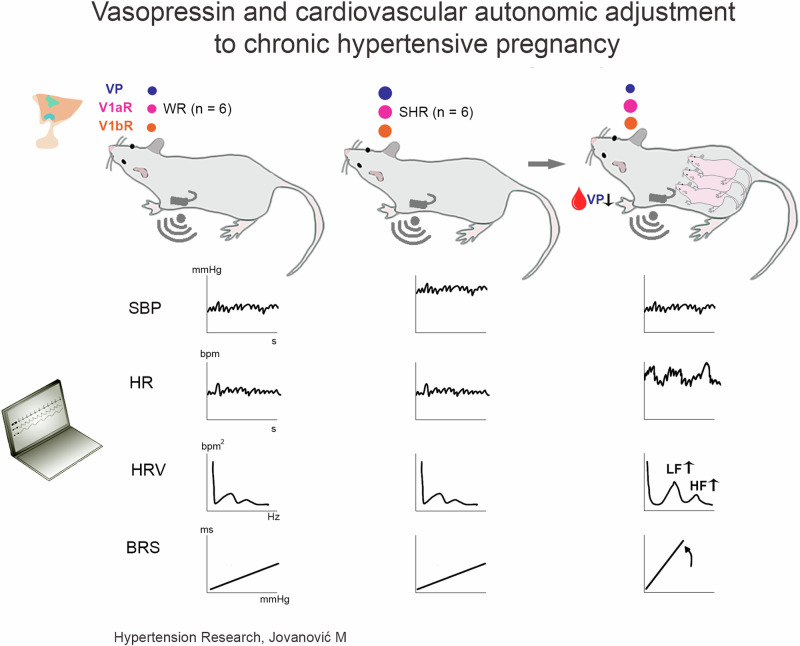
Pregnant SHR dams have reduced expression of VP in SON associated with increased V1bR expression, lower plasma VP, normal BP during late pregnancy and marked signs of enhanced sympathetic cardiac stimulation (increased HR and LFHR variability) and recruitment of vagal mechanisms (enhancement of BRS and HFHR variability).

Pregnant SHR dams have reduced expression of VP in SON associated with increased V1bR expression, lower plasma VP, normal BP during late pregnancy and marked signs of enhanced sympathetic cardiac stimulation (increased HR and LFHR variability) and recruitment of vagal mechanisms (enhancement of BRS and HFHR variability).

## Introduction

Chronic hypertensive pregnancy (CHP) is defined as the rise of systolic blood pressure (SBP) >140 mmHg and/or diastolic blood pressure (DBP) >90 mmHg, observed before the 20th week of gestation [1]. It is estimated that CHP is present in 0.9–1.5% of pregnant women. A further growth in the number of pregnant individuals with CHP is expected due to increasing obesity rate and trends towards and upward shift in the conception age [1, 2].

In CHP, both foetal and maternal complications are increased and may be very serious, including placental abruption, peripartum hemorrhage, cerebrovascular accident, pulmonary edema, and acute kidney injury [2, 3]. Also, later in life, mothers have an increased incidence of cardiovascular, metabolic, and cerebrovascular diseases [1, 3].

In pregnancy, the mother exhibits numerous physiological changes. Most of these modifications start in early pregnancy and are maintained throughout the whole period of gravidity in order to provide enough blood and oxygen supply for the developing foetus, guaranteeing the positive pregnancy outcome [4]. Cardiovascular adaptation to normotensive pregnancy includes vasodilation followed by a decrease in total peripheral resistance and the consequent decrease in arterial blood pressure (BP) and an increase in heart rate (HR) and cardiac output due to enhanced sympathetic outflow. Systemic and renal vasodilation is accomplished by increasing vasodilator synthesis [5, 6] and by reducing vascular sensitivity to vasoconstrictors [7], both of which oppose compensatory sympathetic hyperactivity [8, 9]. In normal pregnancy, increased maternal plasma volume leads to relative hyponatremia and anemia [7, 10]. The expanded circulating blood volume in pregnancy is due to the resetting of the osmotic threshold for vasopressin secretion [11, 12]. Yet, the neuroendocrine and autonomic adaptation of the cardiovascular system to hypertensive pregnancy is insufficiently understood.

It is well established that neurohypophyseal peptide oxytocin plays a key role in neuroendocrine adaptation to healthy normal pregnancy, delivery, and lactation. However, the impact of vasopressin (VP) in the same physiological context is not fully understood. VP and oxytocin are synthesized in magnocellular neurons of supraoptic nucleus (SON) and paraventricular (PVN). They both act as neurohormones and neurotransmitters to modulate the cardiovascular system. Magnocellular neurons in SON and PVN release VP into the circulation where it regulates blood volume and BP. At the same time, parvocellular VP-synthesizing neurons of the PVN project to the rostral part of the medulla (RVLM) where sympathetic outflow to the cardiovascular system is set, as well as to the intermediolateral column of the spinal cord (IML) where the bodies of sympathetic ganglionic neurons are located. From both projections, VP neurons can modulate autonomic control of the cardiovascular system [13–16]. Under baseline physiological conditions, gamma-aminobutyric acid (GABA) containing neurons surrounding the PVN tonically inhibit excitatory glutamate inputs to pre-sympathetic neurons. During stress or pathological dysregulation magnocellular neurons (MCNs) and parvocellular neurons (PCNs) are activated to a different degree by being disinhibited or stimulated by glutamate [17, 18].

In a well-recognized animal model of genetic hypertension—Spontaneously hypertensive rats (SHR) [19, 20] and human hypertensives [21] VP has been shown to play an important pathophysiological role and contribute to the severity of the disease [13, 14]. We thus hypothesized that neuroendocrine adaptation to pregnancy by VP differs in hypertensive and normal pregnancy. Our aim was to quantify gene expression of VP, and its V1aR and V1bR receptors within the PVN and SON in SHRs and control Wistar rats (WR) dams. These data were compared with VP secretion, and with autonomic cardiovascular adaptations to pregnancy in normotensive and hypertensive animals using spectral indices of cardiovascular variability and spontaneous baroreflex sensitivity (BRS).

## Materials and methods

The experimental protocol was approved by the School of Medicine University of Belgrade Ethic Committee review board and The Ministry of Agriculture, Forestry and Water Economy of the Republic of Serbia (license n°119–01–13/17/2015–09). All experimental procedures in this study conform to the guidelines from Directive 2010/63/EU of the European Parliament on the protection of animals used for scientific purposes, as well as the U.K. Animals Act, 1986, and ARRIVE guidelines, National Act on Animal Welfare 2009/6/RS, and Rule book 2010/RS.

### Animals

Experiments were performed on 12 weeks old female WR (*N* = 22) and SHR (*N* = 22) rats (185–250 g) bred at a local animal facility, housed individually in controlled environment (12 h/12 h light dark-cycle, lights on at 7:00 a.m., temperature 21 ± 2 °C and humidity 60 ± 5% [v/v]) with free access to standard pelleted chow (0.2% sodium content, Veterinarski zavod, Subotica) and tap water. Before all experimental procedures, the animals were allowed a 2 week long adjustment period for acclimation to the new environment and animal handling, including sampling of vaginal smears. At the end of the experiment, all rats were euthanized by decapitation.

### Surgical implantation of radio telemetry devices

Virgin female WRs (*N* = 6) and SHRs (*N* = 6) were anaesthetized by combined ketamine (90 mg/kg, i.p.) and xylazine (10 mg/kg, i.p.) anesthesia. After preoperative trichotomy and disinfection of the abdominal area with hydrogen peroxide and povidone-iodine, a 3 cm-long ventral midline incision was made and the intestines were retracted. A catheter tip of the radiotelemetric probe (TA11-PA C40; DSI, Transoma Medical, St Paul, MN, USA) was inserted into the exposed aorta, fixed with 3M VetbondTM and tissue cellulose patch (DSI, Transoma Medical). The body of radiotelemetric probe was placed in the abdominal cavity and attached to the anterior abdominal wall. The surgical incision was closed by two layers of non-absorbable sutures. Postoperative treatment was provided by cleaning the sutured incision with hydrogen peroxide and povidone-iodine. Gentamicin (25 mg/kg, i.m.) was given preoperatively and for the following three days to prevent infection. For pain management, rats received carprofen (5 mg/kg/day, s.c.) on the day of the surgery and for the following two days. The recovery period lasted for 2 weeks including body weight and the estrus cycle [22].

### Determination of the estrous cycle and pregnancy

In order to determine the estrous cycle female rats, Wistar and SHR, were examined daily, approximately for 1 month (2 weeks prior to the surgical implantation procedure, during recovery period and data acquisition). A plastic pipette tip (prefilled with 10 μL 0.9% [w/v] NaCl) was inserted into the atrium of their vagina to collect fluid, a drop of which was placed on glass slides to be observed unstained under a light microscope with magnification lenses 10× and 40×. Three types of cells can be recognized in unstained vaginal smear: epithelial round-shaped cells with nucleus, cornified cells of irregular shape without nucleus, and leukocytes, the smallest round cells. The proportion of these cells in the smear was used to determine the phases of the estrous cycle. According to Marcondes et al. [23, 24], proestrus smear consists of a predominance of epithelial cells; an estrous smear consists of a predominance of cornified cells; while a metestrus smear has the same proportion of leukocytes, cornified, and epithelial cells. Diestrus smear consists of a predominance of leukocytes. At diestrus, female rats were recorded for basal physiological values of cardiovascular hemodynamic parameters before pregnancy. On the night of proestrus, a single virgin female equipped with a transmitter device was housed with a sexually experienced male rat of the same strain. In order to achieve a pregnancy, in the morning, after the couple was caged together, a vaginal smear was re-examined and the presence of spermatozoa or vaginal plug was considered Day 0 of gestation. The average gestation time for WRs is 21 days, while for SHRs is 22 days (data from our local Animal facility).

### Experiment design

SHR and WR females, equipped with TA-11-PAC40 devices were subjected to continuous measurement of cardiovascular hemodynamics. Data acquisition was performed before pregnancy—diestrus, in the middle and late pregnancy (gestational day 20/21). After decapitation, truncal blood and the brain were collected for further analysis.

### BP and HR acquisition and cardiovascular variability analysis

Arterial blood pressure signals were continuously transferred from the radio-telemetry device implanted in rat’s aorta and placed in the intra-abdominal cavity of rats to a computer equipped with Dataquest A.R.T.4.0. software (DSI, Transoma Medical, St. Paul, USA) specialized in the acquisition and analysis of cardiovascular signals. Arterial BP was recorded at 1000 Hz sampling frequency, then resampled at 20 Hz in a Dataquest A.R.T.4.0. software. Systolic pressure, diastolic pressure, and pulse interval or its inverse value (HR) were derived from arterial pulse waves as maximum, minimum, and inter-beats interval, respectively. After the manual withdrawal of artefacts, 410s-long stationary segments were chosen for power spectrum analysis on 30 overlapped 2048-point time series [25, 26]. Power spectra were analyzed in total (TV: 0.00976–3 Hz), very-low-frequency (VLF: 0.00976–0.195 Hz), low-frequency (LF: 0.195–0.8 Hz) and high-frequency (HF:0,8–3 Hz) range. LF-BP and LF/HF-HR are recognized markers of sympathetic outflow to arterial blood vessels and sympatho-vagal balance to the heart in both experimental [27, 28] and clinical practices [25, 26].

### BRR evaluation by sequence method

The spontaneous baroreceptor reflex sequence is a stream of consecutively increasing/decreasing SBP samples, followed by a stream of increasing/decreasing pulse interval samples delayed by 3, 4, or 5 beats regarding SBP. A threshold for sequence length was set to four beats. The sensitivity of baro-receptor reflex [ms/mmHg] was assessed as a linear regression coefficient averaged over all identified sequences, baro-receptor reflex sensitivity (BRS) = (pulse interval-const)/(systolic blood pressure) [29]. A more refined method for this dependency analysis is proposed in Jovanovic et al. [30].

### Tissue and blood collection

Additional four groups of females, diestrus WRs (*N* = 8) and diestrus SHR (*N* = 8), and late pregnant WRs (*N* = 8) and SHRs (*N* = 8), previously time-mated in our research facility, were euthanized by decapitation (Rat guillotine, Harvard Apparatus, Holliston, MA, USA). The brains were harvested, frozen in liquid nitrogen, and stored at −80 °C for PVN and SON sampling. The blood samples were taken according to the manufacturer’s instructions and stored at −80 °C for VP plasma estimation.

### Evaluation of mRNA expression of VP, V1aR and V1bR in the rat PVN and SON

By using a cryostat (Leica Microsystems CM1900, Leica Microsystems, Nussloch GmbH, Nussloch, Germany), 60 μm thick caudal-rostral brain slices were taken and stained with Toluidine blue (1% [w/v] in 70% [v/v] ethanol, Sigma-Aldrich Co. Ltd, Poole, Dorset, UK) for mapping and verification of the hypothalamus. Both bilateral SON and PVN, unstained tissue was punched, taking 1 mm in diameter, using a 15-G Sample Corer (Fine Science Tools Inc., Foster City, CA, USA, cat.no 18035-01), and stored in RNase-free tubes (ISOLAB Laborgeräte GmbH, Eschau, Germany) at −80 °C.

### RNA extraction, cDNA synthesis, and real time quantitative polymerase chain reaction (qPCR) and data analysis

Total RNA was extracted from samples by TRIzol reagent (Life Technologies, Carlsbad, USA), chloroform (Sigma-Aldrich, Taufkirchen, Germany), and 2-propanol (Sigma-Aldrich, Taufkirchen, Germany). RNA quality and yield were assessed by using BioSpec-nano (Shimadzu, Kyoto, Japan). Thus, cDNA was synthesized by QuantiTect Reverse Transcription Kit (Qiagen, Hilden, Germany) according to the manufacturer´s instructions. RT-qPCR was obtained in Applied Biosystems ViiaTM 7 (Foster City, CA, USA). All assays were performed in triplicates, using 96-well reaction plates (Foster City, CA, USA). The reaction volume contained: 1 µL cDNA sample, 0.048 µL 100 nM primers (Invitrogen, Pacle, UK), 6 µL Power SYBR Green PCR Master Mix (Applied Biosystems, Warrington, UK), and 4.9 µL RNase-free water (Qiagen, Hilden, Germany). The Primer Express software (version 3.0.1, Thermo Fisher Scientific, Foster City, CA, USA) and GenBank database were used to design specific primers for: vasopressin gene (forward: 5′-GCA CCC ATC AGC CTA ATT CG-3′; reverse: 5′-CGC CAA CCT ATT ATG CCC TAG TA-3′), V1aR (Rn Avpr1a 1 SG QuantiTect Primer Assay, QT00402990, NM053019), V1bR (forward: 5′-TGCCACATTCCTGGAGTACCT-3′; reverse: 5′-AGGACGGTTAACCAAGTAG TGAGATG-3′) and Rpl19 gene (forward: 5′-GCG TCT GCA GCC ATG-3′; reverse: 5′-TGG CAT TGG CGA TTT-3′) as a house-keeping gene. The applied RT-qPCR reaction was composed of cDNA denaturation (50 °C–2 min, 95 °C–10 min), 40 cycles of cDNA amplification (95 °C–10 min, 60 °C–1 min), and melting curves analysis (95 °C–15 s, 60 °C–1 min, 95 °C–15 s). Relative gene expression was estimated by the 2-ΔΔCt method [31].

### Determination of VP plasma level

Truncal blood samples were collected in sterile plastic tubes using ethylenediaminetetraacetic acid as an anticoagulant. The samples were centrifuged for 15 min at 1000 × *g* at 4 °C within 30 min of collection. The obtained plasma samples were then aliquoted in sterile plastic tubes and kept at −80 °C until analysis. Vasopressin plasma levels were quantified by enzyme-linked immunosorbent assay (ELISA) method using Rat ADH (Antidiuretic Hormone) ELISA Kit (Elabscience Biotechnology Inc., USA) with a sensitivity of 9.38 pg/ml. The assay was performed according to the manufacturer’s instructions. The optical density was determined using a microplate reader set to 450 nm (Multiskan EX, Thermo Electron Corporation, Vantaa, Finland). Vasopressin plasma levels were expressed in pg/ml.

### Statistical analysis

Data are represented as median, minimum, and maximum values. Data were analyzed using IBM SPSS software (SPSS Statistics v.20 software IBM Corporation, NY, USA). Data distribution was examined using the Kolmogorov–Smirnov test. For each experimental group, cardiovascular parameters collected over time, on diestrus, in middle and late pregnancy were evaluated by the Independent Friedman test. Data are represented as median, minimum and maximum values. qPCR data and VP plasma level data were analyzed by the Kruskal–Wallis test (followed by the Wilcoxon test). Statistical significance was considered at *p* < 0.05.

## Results

### VP and VP receptor gene expression in the PVN and the SON of non-pregnant and pregnant rats

**Fig. 1:**
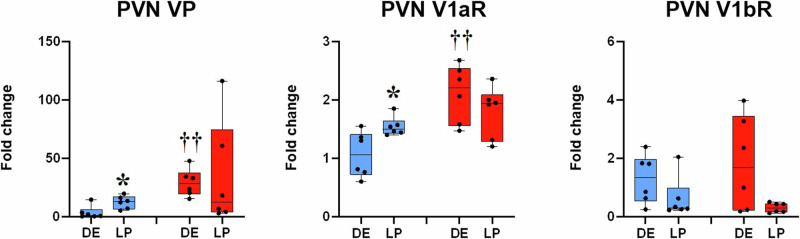
VP and VP receptors gene expression in the PVN of diestrus and late pregnant WRs and SHRs. SHRs exhibit upregulation of VP and V1aR mRNAs in diestrus and are maintained until the end of pregnancy. A notable increase of VP and V1aR mRNA expression can be observed in the PVN of late-pregnant WRs. Blue boxes: WR female rats; red boxes: SHR female rats; DE diestrus; LP late pregnancy. Data were analysed using Kruskal–Wallis followed by Mann–Whitney U statistical tests and significance was *p < 0.05 vs diestrus; ††*p* < 0.05 vs WR

Before pregnancy, SHR females showed elevated VP and V1a gene expression in the PVN compared to WR counterparts, and their levels persisted throughout gestation. In late pregnancy, VP and V1a receptor gene expression in the PVN increased in WRs in respect to diestrus, becoming comparable to SHR. V1bR mRNA levels were not statistically different between strains before or during pregnancy (Fig. 1).

**Fig. 2:**
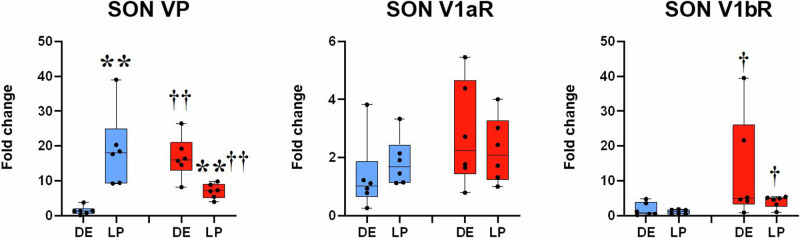
VP and VP receptors gene expression in the SON of diestrus and late pregnant WRs and SHRs. A marked pregnancy-induced increase of VP can be noted in the WR group. On the contrary, pregnancy decreased the initially higher gene expression of VP in SON of SHRs. V1bR mRNA was elevated and remained elevated in pregnancy in SHR compared to WRs. Blue boxes: WR female rats; red boxes: SHR female rats; DE diestrus; LP late pregnancy. Kruskal–Wallis followed by Mann–Whitney test was performed with significance **p < 0.01 vs diestrus; †*p* < 0.05 vs WR; ††*p* < 0.05 vs WR

In the SON of non-pregnant SHR, VP mRNA and V1b mRNA were increased in respect to non-pregnant WRs. Pregnancy differentially affected VP expression in strains: VP gene expression was increased in WRs and decreased in SHRs. The expression of V1bR was not altered by pregnancy in both strains (Fig. 2).

### Plasma VP levels in non-pregnant and pregnant rats

**Fig. 3:**
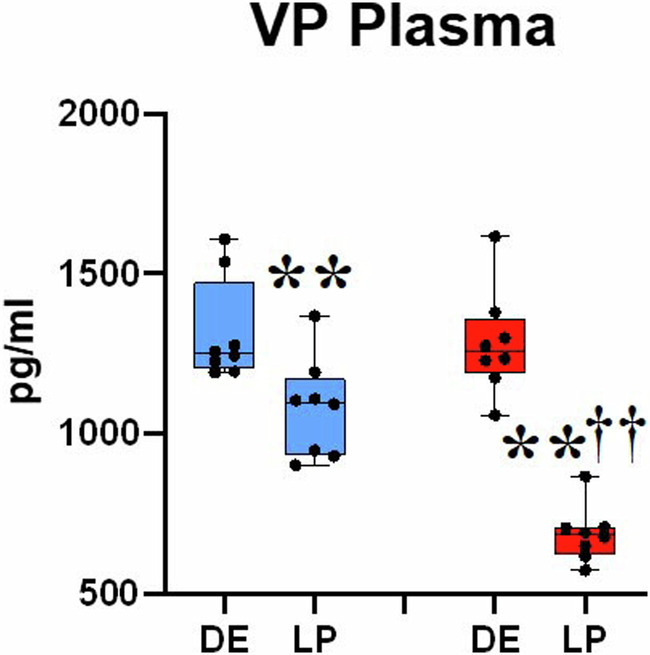
Plasma VP of diestrus and late pregnant WRs and SHRs. A marked diminution of plasma VP levels induced by pregnancy was seen in both experimental groups, with a more profound change in SHRs at the end of the pregnancy. Blue boxes: WR female rats; red boxes: SHR female rats; DE diestrus; LP late pregnancy. The data were estimated by Kruskall–Wallis test and Mann–Whitney statistical test; **p < 0.01 vs diestrus; ††*p* < 0.05 vs WR

Plasma VP concentrations were the same in both strains before pregnancy. In both normotensive and hypertensive females, pregnancy reduced plasma VP levels, with a more prominent drop in late pregnant SHRs (Fig. 3).

### Cardiovascular hemodynamics and spectral markers in non-pregnant and pregnant rats

**Fig. 4:**
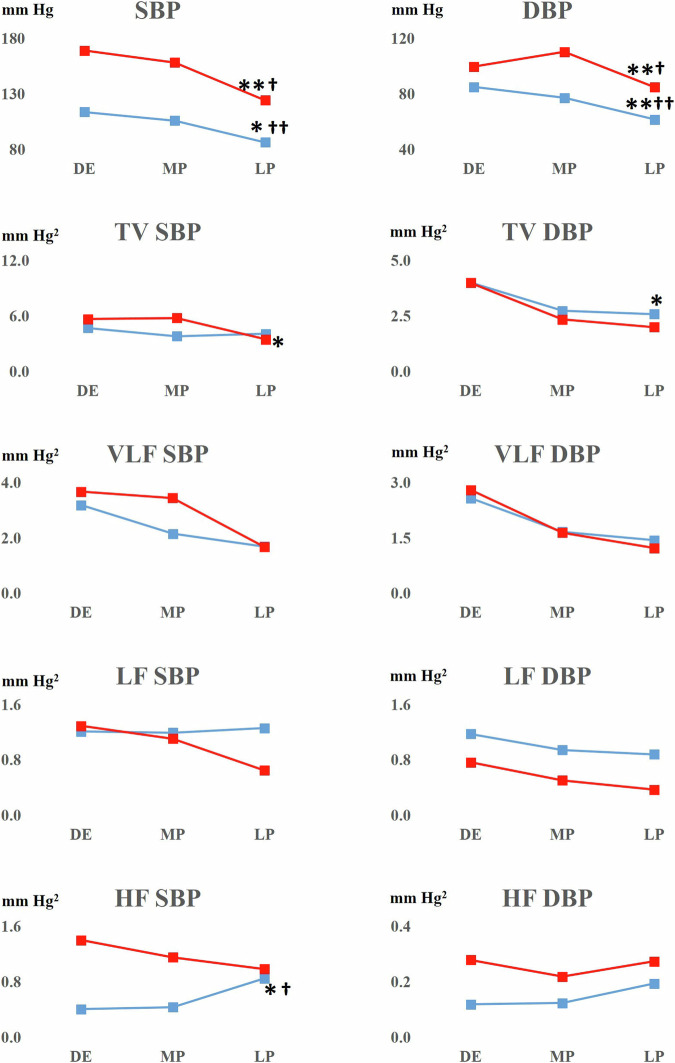
SBP and DBP short-term variability in WR and SHR rats. Pregnancy triggers a marked decrease of SBP and DBP in both groups, with total BP variability downfall in respective groups. Also, late-pregnant WRs demonstrated an increase of HF SBP. Blue lines: WR female rats; red lines: SHR female rats; DE diestrus; MD mid-pregnancy; LP late pregnancy. Data were analysed using the independent Friedman test and significance was *p < 0.05 over time; **p < 0.01 over time; †*p* < 0.05 late pregnancy vs diestrus baseline; ††*p* < 0.01 late pregnancy vs diestrus baseline

Regardless of the different starting points of SBP and DBP in non-pregnant WRs and SHRs over the course, pregnancy decreased both SBP and DBP. The decrease of SBP and DBP was pronounced in SHR and reached normal normotensive values in late pregnancy. The reduction of SBP and DBP was followed by a reduction of total SBP and DBP variability. The spectral analysis also revealed an increase of HF SBP domain in pregnant WRs (Fig. 4).

**Fig. 5:**
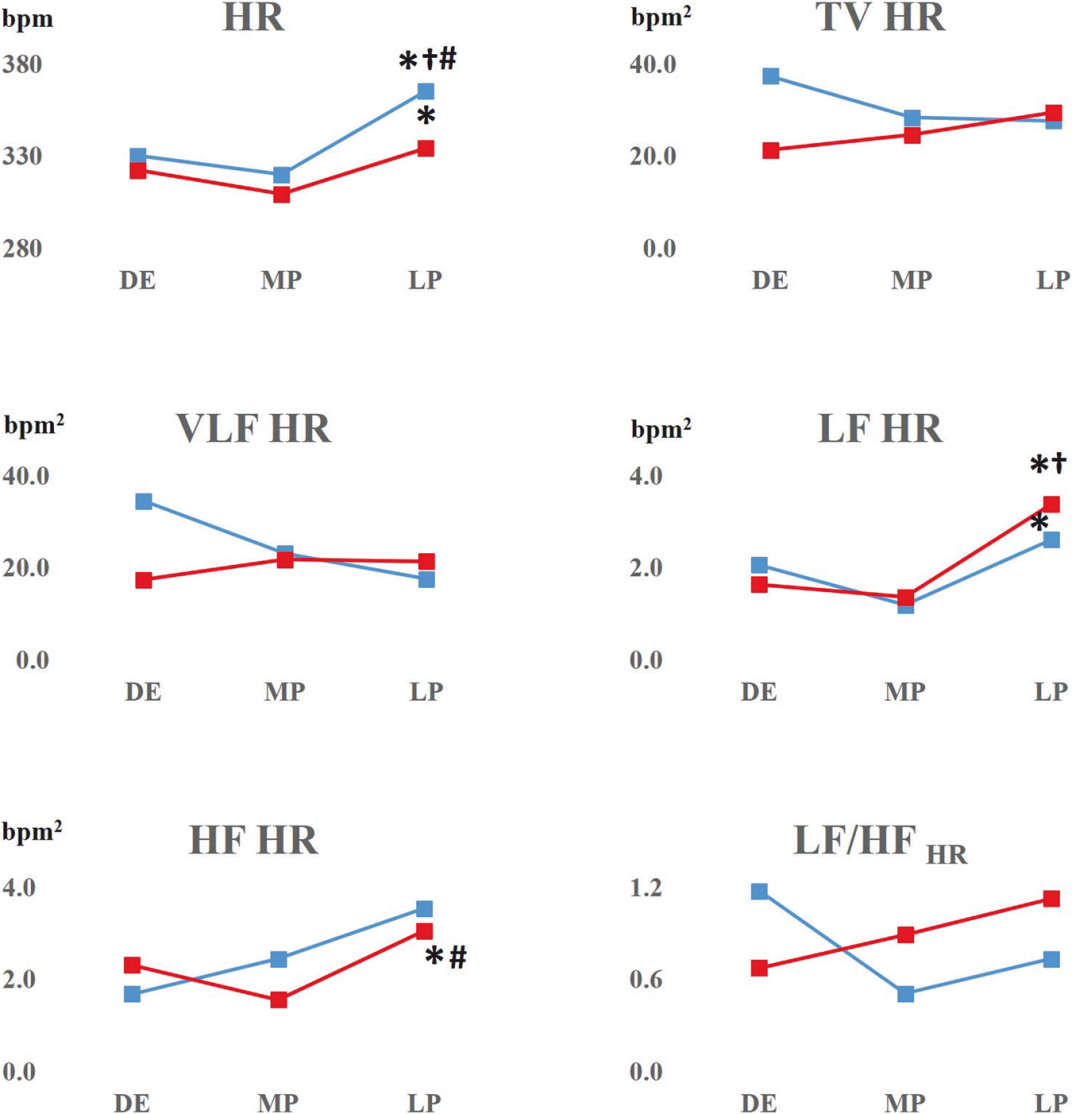
HR and HR short-term variability in WR and SHR rats. Note the increase of HR throughout the course of pregnancy in both strains. SHRs exhibit marked uprise in LF and HF-HR band, without significant change in LF/HF ratio. Blue lines: WR female rats; red lines: SHR female rats; DE diestrus; MD mid-pregnancy; LP late pregnancy. The data were estimated by independent Friedman test; *p < 0.05 over time; †*p* < 0.05 late pregnancy vs diestrus baseline; #*p* < 0.05 late pregnancy vs middle pregnancy

HR increased in both strains at the end of pregnancy (Fig. 5). In SHR and WRs, LF-HR also increased. This increase was greater in SHR denoting marked sympathetic cardiac stimulation. However, in SHR and WRs a marker of sympathovagal balance LF/HF-HR remained unchanged. This was due to a concomitant increase of vagally mediated HF-HR. (Fig. 5).

### Baroreceptor reflex sensitivity in non-pregnant and pregnant rats

**Fig. 6:**
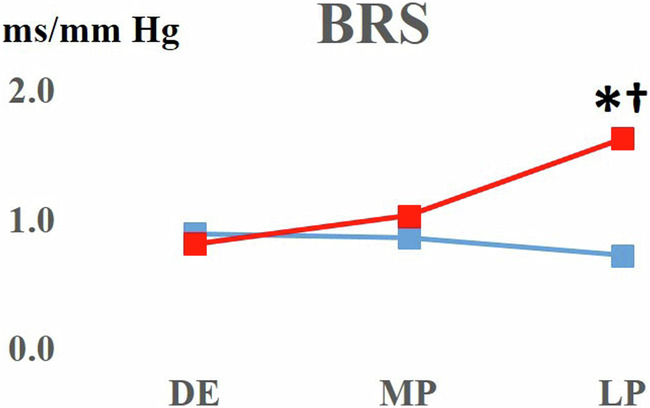
BRS in WR and SHR rats. SHRs exhibit a rise in BRS as the pregnancy approaches late stage. Blue lines: WR female rats; red lines: SHR female rats; DE diestrus; MD mid-pregnancy; LP late pregnancy. The independent Friedman test was performed for data regarding BRS; *p < 0.05 over time; †*p* < 0.05 late pregnancy vs diestrus baseline

The baroreceptor reflex sensitivity did not change in WRs over the course of pregnancy. However, in SHR dams, BRS increased significantly in late pregnancy with respect to diestrus SHR values and pregnant WRs (Fig. 6).

## Discussion

This study shows, for the first time, that whilst pregnancy increases VP gene expression in the PVN and SON of normotensive WR, it decreases in the SON of pregnant SHRs resulting in a considerable drop in plasma VP concentration. In SHR rats before and throughout pregnancy V1aR and V1bR are over-expressed in PVN and SON respectively, while in Wistar dams the expression of V1aR in PVN increases only in late pregnancy. PVN V1aRs expressed on PCNs, can be activated by intranuclearly released VP and enhance sympathetic outflow to the heart [32], as observed in both pregnant WRs and SHRs. Also, V1aR and V1bR on MCNs have been shown to orchestrate VP release [33]. It seems that in SON V1bR outweighs autocontrol management of VP synthesis in MCNs [34].

The drop of plasma VP was associated with BP normalization in SHR in late pregnancy, recruitment of baroreflex mechanism leading to marked tachycardia, and LF-HR variability uncovering cardiovascular vulnerability of pregnant SHR regardless of BP normalization.

It is thought that VP is involved in the etiology of hypertension [35]. In this context, both the hormonal and neurotransmitter functions of VP are considered to contribute to the development of this condition [13, 36, 37]. SHRs and hypertensive humans have elevated plasma VP levels and increased vasculature sensitivity to exogenously applied VP, corroborating with the severity of clinical presentation [36, 38]. Brain VP content in the stroke-prone variety of SHRs is upregulated in PVN and SON with the progression of hypertension [39]. We have also shown that Borderline hypertensive rats have greater basal expression of VP in the PVN, inherited from their hypertensive parent (SHR mothers) [40]. According to available research data, VP plasma content of SHRs should correlatively increase with the progression of hypertension. A detectable rise in circulatory VP can be observed starting from the 24th week of age (six months old) in SHRs, whilst in the prior period, VP circulatory levels are the same in normotensive and hypertensive rats [41]. This is in line with our finding of similar VP plasma levels in 3-month-old SHR and WR dams during diestrus. Our results support the concept of heightened VP system activity in the hypothalamus of SHR females before pregnancy. SHR dams have increased VP gene expression within the PVN and SON in the diestrus phase with respect to WRs, possibly as a result if increased VP gene transcription.

An interesting finding is that pregnant SH rats exhibited a marked decrease of VP gene expression in the SON and a marked decrease in plasma VP concentrations. A similar finding was reported in women where the decrease of VP in pregnancy is ascribed to increased clearance by placental peptidases [42]. However, the placenta of pregnant rats do not apparently synthesize vasopressinase [43], so in the present study, the decrease of plasma VP in pregnancy is rather due to reduced release of VP into plasma rather than increased plasma clearance. In this context, it is important to mention that hypoosmolality during pregnancy does not affect VP release as it would be expected, due to the resetting of MCNs [11, 42]. Surprisingly, VP does not seem to be the underlying mechanism for AQP2 over-expression in renal collecting ducts and pregnancy-induced water overload [44]. A possible culprit for the retained VP secretion into circulation observed in our data could be relaxin. It has been shown in gravid Sprague Dawley rats that VP release is relaxin mediate [45]. The same study reports basally lower VP plasma levels in 21-day pregnant rats compared to non-pregnant animals. Also, the effects of relaxin-orchestrated VP secretion seem to wither overtime, and are attenuated in the late pregnancy stage, supporting the minimal VP plasma at the end of the pregnancy measured in our experiments [45]. The decrease of plasma VP concentration could contribute to the decrease in BP by lack of the stimulation of V1aR in smooth muscle cells which contribute to overall peripheral resistance and thus BP level.

Apart from being secreted into the circulation to act as a hormone, VP is also being released from axons of PCNs (pre-autonomic neurons located in the parvocellular part of the PVN) into the RVLM and IML of the spinal cord where presympathetic neurons and preganglionic sympathetic neurons are located to increase sympathetic outflow to the cardiovascular system [15]. Almost a quarter of these parvocellular projecting neurons synthesise VP [46] and V1aRs are abundantly expressed in both RVLM and IML [47, 48]. Under basal physiological conditions, NO tonically inhibits neuronal activity in hypothalamic magnocellular and pre-autonomic neurons mainly due to presynaptic facilitation of GABA-ergic neurotransmission [49–51]. In hypertension, glutamatergic activation of the PVN in the SHR drives elevated basal sympathetic activity [52]. In addition, in normal pregnancy, NO production in PVN is decreased and PVN neurons are disinhibited enhancing sympathetic outflow to the periphery [53]. Our findings corroborate the view of increased sympathetic activity to the heart of pregnant Wistar damns, and, strikingly to the hearts of SHR pregnant dams, as denoted by increased HR and LF-HR spectral parameters.

An increase of vasopressin V1aR in PVN and V1bR mRNAs in SON of SHR dams needs further elaboration. Wistar pregnant rats manifest increased expression of V1aR and VP in SON in late pregnancy. V1aR and V1bR were found in abundance on MCNs [33, 40, 54] to exert autocrine control on the somatodendritic release of VP. Somatodendritic release of VP orchestrates the firing rates of MCNs to stimuli, such as blood osmotic status, volume, and pressure to best fit the physiological demands [55]. The spillover of somatodendritically released VP within the PVN is proven to excite spinally projecting parvocellular neurons by activation of V1aR residing on their membrane, resulting in coordinated neuroendocrine and sympathetic response to homeostatic challenges [32]. A somatodendritic discharge of VP is separately regulated from the plasma VP release and it may occur singly [56]. The content of vesicles carrying VP intended for a somatodendritic release differs from the ones destined for axonal transport, and are enriched with galanin [57]. There is no clear view on VP receptor expression in PVN during pregnancy, and only few studies referred to this issue. It seems that the V1aR distribution is rather stable, with most dynamic changes occurring in postpartum and during lactation. Nevertheless, it has been shown that V1aR is upregulated in the PVN of prairie voles in late pregnancy stages, indicating maternal priming of the PVN for V1aR-mediated sensitivity to VP [58, 59]. Our results coincide with this finding and report elevated V1aR mRNA levels along with increased VP expression, of both strains in late pregnancy. We can speculate that V1aR located on PCNs that project to the RVLM and IML could act to increase sympathetic outflow to the heart as a response to generalized vasodilation in late pregnancy in both strains. However, during late pregnancy in the SON of SHR, the expression and release of VP was reduced while V1bR expression remained increased in respect to WR. Lately, it has been suggested that V1bR might be mainly responsible for the VP autoregulation of magnocellular neurons in SON, providing a short-loop feedback mechanism that affects VP synthesis locally [34, 60]. Our results indicate recruitment of these autocrine mechanisms on MCNs in SHR mitigating negative feedback on VP synthesis to counteract markedly reduced VP blood levels. Autocrine roles of VP receptors on VP synthesis in MCNs probably underlies differences between strains in late pregnancy. For instance, V1bR over-expression in the SON of SHR decreases VP mRNA expression while the lack of it in WR enhances it. Also V1aR over-expression in the PVN of WR in late pregnancy may be the cause of boosted VP expression in WR which annuls species differences in VP synthesis in late pregnancy.

As expected, late pregnancy reduced SBP and DBP and their variabilities, followed by reflex increase of HR in both strains. These findings are in agreement with the results of previously conducted studies in animals [61, 62]. Apart from the well-described vasodilation and compensatory sympathetic increase, these hemodynamic data might be justified in correlation with the VP plasma decrease observed during pregnancy in our animals. However, a more noticeable drop in BP was observed in SHR pregnant females, corresponding to even lower VP values measured in the plasma of hypertensive animals, which is no surprise since VP is a potent pressor agent [36].

According to our results, the reduction in BP and BP TV observed in both WRs and SHRs occurs mainly due to a decrease in VLF-BP range and can be explained by over-expression and synthesis of AT2R in blood vessels of pregnant rats which mediate vasodilation [63], decrease in plasma VP, increased synthesis of vasodilating molecules [5, 6] and blood vessels indifference to vasoconstrictors [7]. Interestingly, LF-BP variability tended to decrease and did not reflect increased sympathetic tone directed to blood vessels due to vascular indifference to noradrenaline. Normotensive pregnancy is usually stated as only ‘healthy’ physiological conditions of basal sympathetic hyperactivity [8]. However, pregnancy is also characterized by impaired neurovascular transduction [62, 64], meaning blood vessels are unresponsive to sympathetic stimulation, supporting a sustainable decrease in total peripheral resistance [65–67]. Of note is that WRs displayed an increase of HF SBP with the gestational progress, described previously by others [61]. The HF zone of SBP variability may be elevated by profound vasodilatation [68] or by changes in breathing patterns [69]. Since WR dams in our experiments did not exhibit any change in the respiratory rate advocated by HF position in BP and HR spectra, change in HF SBP can be attributed to pregnancy-induced vasodilatation and consequent thoracic blood vessels under-filling.

Cardiac indices of increased sympathetic stimulation during late pregnancy were obvious in both strains and documented by increased HR and LF-HR variability bands, and were especially marked in SHR dams. At the same time, SHR dams had increased vagally mediated HF-HR variability. The increase of vagal influences to the heart is reported to be useful in protecting the heart against sympathetic overstimulation, involving cholinergic NO synthesis in ventricles, and may improve cardiac health issues [70]. Altogether, increased LF-HR and HF-HR along with no change in LF/HF index, suggest that both autonomic limbs are co-active at the same time and lead to efficient cardiac function in pregnant SHRs [71]. Perhaps the increase in vagal tone to the heart could be mediated by centrally acting vasopressin and the stimulation of V1aR in the medulla [72]. An increase in BRS in SHR in late pregnancy also denotes enhanced vagal drive to the heart. Only a few papers have considered autonomic adjustment of cardiovascular control in CHP with contradictory reports. Some report a decrease in arterial blood pressure accompanied by unchanged BRS in pregnant SHR [62], reduced BRS [73], or increased BRS [74] in chronic hypertensive women [73]. Enhanced parasympathetic cardiac modulation in SHRs was also reported to be improved [62].

It would be of interest to evaluate and compare the effects of antihypertensive drugs on plasma VP, BRS, and HRV as well as associated risks for mother and child in CHP. One of the reasons why non/treated subjects with CHP, even though with normalized blood pressure, are prone to hypertensive crisis, maybe due to sympathetic overstimulation that overcomes protective vasodilative mechanisms associated with pregnancy. Also, hypervolemia predisposed to blood pressure increase. Therefore, strong vasodilators are necessary to prevent/treat hypertensive crises. According to revised ACOG guidance (2019) based on the CHAP trial [75] treatment of mild CHP is recommended as well as tight control of BP in severe cases.

**In conclusion**, our results show that pregnant SHR dams have reduced expression of VP in SON associated with increased V1bR expression, lower plasma VP, and normal BP during late pregnancy. They also show marked signs of enhanced sympathetic cardiac stimulation (increased HR and LF-HR variability) and recruitment of vagal mechanisms (enhancement of BRS and HF-HR variability) to defend against it.
